# Dietary and Nutritional Aspects of Metabolic Syndrome Management: An Overview

**DOI:** 10.2174/0118715303316445241108100017

**Published:** 2025-01-14

**Authors:** Pargat Singh, Ujjwal Kaushik, Showkat R. Mir, Neha Kukreti, Sharad Visht

**Affiliations:** 1 Chitkara College of Pharmacy, Chitkara University, Rajpura, Punjab, India;; 2 Department of Pharmacognosy, School of Pharmaceutical Education and Research, Jamia Hamdard, New Delhi 110062, India;; 3 Department of Pharmacy, Tishk International University, Erbil, Kurdistan Region, Iraq

**Keywords:** Metabolic syndrome, dietary pattern, mediterranean diet, nutrients, physical activity, ketogenic diet

## Abstract

Sedentary lifestyles and prolonged physical inactivity are often linked to poor mental and physical health as well as an increased risk of a number of chronic illnesses, including cancer, obesity, type 2 diabetes, and cardiovascular problems. Metabolic Syndrome (MetS), as the new disease, has emerged as the world's leading cause of illness. Despite having its roots in the West, this issue has now completely globalized due to the development of the Western way of life throughout the world. It currently affects almost one-fifth of the American and European populations, and its incidence has increased in Southeast Asian nations as well. Comparing patients with metabolic syndrome to the general population, it is estimated that they have a 5-fold greater risk of diabetes mellitus and a 2-fold increased risk of atherosclerotic cardiovascular illnesses. MetS is a chronic or prevalent condition associated with various lifestyle conditions characterized by abdominal obesity, low HDL-c cholesterol, insulin resistance, high blood pressure, and dyslipidemia. It has been suggested that insulin resistance, chronic inflammation, and neurohormonal activation are the factors behind the development of metabolic syndrome. In lieu of an upsurge in the complications associated with MetS in modern society, many alternative approaches apart from medicine are being constantly explored. Effects of vivid dietary patterns and nutritional interventions have been thoroughly researched, although the most effective dietary approach remains undetermined. This review discussed different etiological aspects of MetS and brought forth the role of nutritional approaches, micro- and macronutrient intake, lifestyle changes, and herbal intervention in its management.

## 1 INTRODUCTION

Due to the sedentary lifestyle and work culture, metabolic syndrome has risen exponentially worldwide. Though the Western world (USA, Europe) is more affected by this syndrome, its prevalence has gained impetus in Southeast Asian countries as well. The first internationally recognized definition of metabolic syndrome was given by the World Health Organisation in the year 1998 as “incidence of insulin resistance (reduced fasting glucose, decreased glucose tolerance or type 2 diabetes mellitus) in addition to two of the following risk factors: hyperlipidaemia (increased triglycerides and low high-density lipoproteins), obesity (waist-hip ratio or body mass index)”.Though the exact cause and etiological cascade are complex to determine, the various risk factors contributing to the development of metabolic syndrome include consumption of a diet rich in saturated fats, excess alcohol consumption, inadequate sleep, smoking, and overstress. The proposed mechanisms contributing to the development of metabolic syndrome include insulin resistance, chronic inflammation, and neuro-hormonal activation.

## 2 PATHOPHYSIOLOGICAL ASPECTS OF METAB-OLIC SYNDROME

**Fig. (1):**
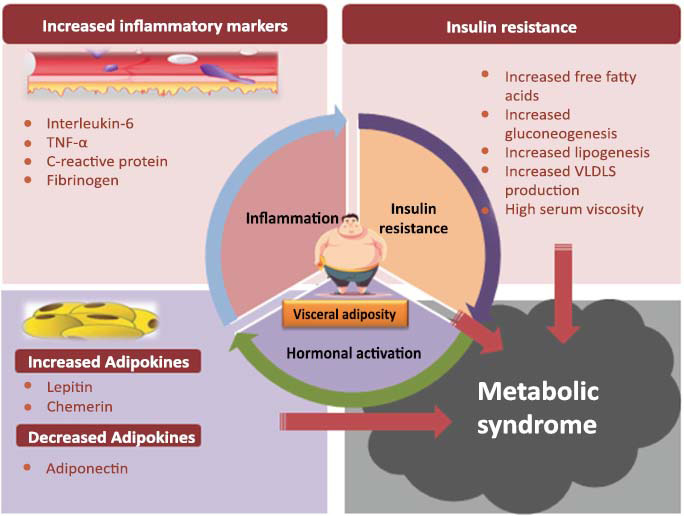
Pathophysiologic aspects related to metabolic syndrome.

It has been documented that sedentary behavior, including obese genetic predisposition, is harmful to the pathophysiology of Metabolic syndrome. The various risk factors contributing to the development of metabolic syndrome include consumption of a diet rich in saturated fats, excess alcohol consumption, inadequate sleep, smoking, and overstress [1, 2]. Though the exact cause and etiological cascade are complex to determine, the proposed mechanisms contributing to the development of metabolic syndrome include insulin resistance, chronic inflammation, and neuro-hormonal activation (Fig. **1**).

### 2.1 Role of Insulin and its Resistance

Insulin increases the liver's and muscles' absorption of glucose while suppressing lipolysis and hepatic gluconeogenesis. A major risk factor for the onset and progression of many severe chronic illnesses is insulin resistance. Changes in metabolic processes and anomalies like inflammation are among the causes of irregularities in the insulin signalling system, which ultimately lead to insulin resistance in the host [3, 4]. Cardiovascular dysfunction and the collection of metabolic disorders are strongly correlated. A key role in the pathophysiology of metabolic syndrome is thought to be played by the rise in circulating free fatty acids *via* insulin resistance. Insulin resistance reduces the breakdown of fat cells, raising levels of free fatty acids. These levels then inhibit muscle protein kinase from activating, which lowers the uptake of glucose. However, free fatty acids in circulation cause the liver's protein kinase to become more activated, which in turn promotes the processes of gluconeogenesis and lipogenesis. A hyper-insulinemic condition is created as a result. Reduced insulin secretion results from a progressive failure of the compensatory mechanism. Insulin resistance contributes to the development of hypertension as well because it impairs the vasodilator effect of insulin and causes vasoconstriction as a result of free fatty acids. Insulin resistance raises the risk of cardiovascular illnesses by releasing pro-inflammatory cytokines from adipose tissue, increasing serum viscosity, and inducing a prothrombotic state.

### 2.2 Role of Inflammation

The role of chronic inflammation in various body parts, including the muscle, liver, gastrointestinal tract, central nervous system, adipose tissue, and pancreatic islets has been related to metabolic syndrome [5]. When obesity is present, the pancreas, liver, muscles, and adipose tissue can all become inflammatory sites. The anti-inflammatory cell population in these tissues changes to a pro-inflammatory one, and this shift is associated with the invasion of immune cells such as macrophages. Systemic inflammatory markers have been well-known for the development of type II diabetes [6]. Inflammatory markers such as TNFα, C-reactive protein (CRP), and IL-6 are elevated in people with MetS [7].

#### 2.2.1 Interleukin-6

Interleukin-6, a pleiotropic inflammatory cytokine, is a crucial indicator for atherosclerotic processes, as it is found in various tissues and cells, including endothelium, adipocytes, and leukocytes [8]. The pro-inflammatory cytokine interleukin-6 (IL-6) released from macrophages and adipocytes regulates differentiation, migration, proliferation, and cell death to create inflammation, which in turn significantly contributes to the pathophysiology of type II diabetes mellitus and insulin resistance [9]. Raised levels of IL-6, IL-8, TNF-alpha, and C-reactive protein in response to systemic inflammation are related to a greater risk of venous thromboembolism [10]. IL-6 increases the hepatic synthesis of acute phase reactants like CRP, stimulates the RAS pathway, and increases vascular cell adhesion molecules (VCAMs) production, leading to prothrombotic conditions and atherosclerosis of arterial walls. Research confirms higher C-reactive protein (CRP) levels in MetS patients are correlated with insulin resistance, blood pressure, poor HDL, triglycerides, and proinflammatory cytokine levels, including TNF and IL-6 [11].

#### 2.2.2 Tissue Necrosis Factor

The synthesis of TNF-alpha is aided by adipose tissue, which also raises soluble TNF- α receptor levels CRP and IL-6, IL-1 receptor antagonist. It has been proposed that TNF- α, not IL-6, is the primary cause of insulin resistance and dyslipidemia and that IL-6 is its marker. Overall, TNF-α (a pro-inflammatory cytokine) is a key player in the inflammatory pathways that drive insulin resistance, adipose tissue dysfunction, endothelial damage, and lipid dysregulation, all of which are central to the development of metabolic syndrome. Therapeutic strategies targeting TNF-α (such as TNF inhibitors) are being explored for managing aspects of this syndrome. Modulation of TNF-α expression impairs insulin signaling by inducing phosphorylation of insulin receptor substrate (IRS) proteins, which hinders glucose uptake by cells. This process contributes to insulin resistance, a hallmark of metabolic syndrome [12]. Prolonged exposure of pancreatic β-cell to pro-inflammatory cytokines such as IL-1β, TNF-α, and IFN-γ results in a decreased capacity of pancreatic beta-cell function to produce and release insulin, resulting in worsening the glucose homeostasis [13]. Obesity is a state of chronic low-grade systemic inflammation which is often associated with metabolic syndrome. In this, there is an increase in adipose tissue macrophages, which secrete high levels of TNF-α. TNFα treatment induces NLRP3 expression and caspase-1 activity in adipocytes, partly through autophagy dysregulation. The activated adipocyte NLRP3 inflammasome participates in mitochondrial dysfunction and insulin resistance. The chronic inflammation in adipose tissue triggered by TNF-α leads to an increase in free fatty acids, further exacerbating insulin resistance and lipid dysregulation [14]. Endothelial dysfunction (ED) is characterized by imbalanced vasodilation and vasoconstriction, elevated reactive oxygen species (ROS), and inflammatory factors, as well as a deficiency of nitric oxide (NO) bioavailability. It is reported that TNF-α enhances the expression of iNOS and TXNIP, and the level of reactive oxygen species (ROS) facilitates the entry of nuclear factor-κB (NF-κB) into the nucleus and promotes injury in human umbilical vein endothelial cells (HUVECs) [15].

### 2.3 Neurohormonal Activation

Steroid hormones, growth hormones, and the hormones secreted by fat tissue play significant roles in the development of metabolic syndrome. There has been evidence linking hormones to alterations in the body's metabolism, even though more studies are required to determine which hormones cause metabolic syndrome and how. Human conditions, including metabolic syndrome, diabetes, and cardiomyopathy, can be brought on by extended adrenal gland cortisol release as a result of chronic stress [16-18]. The prevalence of metabolic syndrome and the age-related decline in testosterone levels have been found to be inversely correlated. Meta-analysis reported that compared to controls, individuals with incident metabolic syndrome had substantially lower baseline testosterone levels [19]. Increased androgens (male sex hormones) are observed in women with polycystic ovarian syndrome (PCOS). Women with PCOS have an amplified risk of metabolic syndrome, which is related to obesity and metabolic features [20].

## 3 ROLE OF TRACE ELEMENTS IN METABOLIC SYNDROME MANAGEMENT

A trace element imbalance has been proposed as a separate risk factor for MetS because it affects the structure of enzymes, proteins, and complex carbohydrates. It has been demonstrated that there is a molecular connection between metabolic homeostasis, and trace elements and peroxisome proliferator-activated receptors (PPARs) have emerged as important regulators connecting them. This is due to the fact that PPARs both actively participate in a number of metabolic processes, including insulin sensitivity and abdominal obesity, and they also react strongly to variations in trace elements [21].

### 3.1 Selenium (Se)

The World Health Organization refers to selenium as “The miracle element of life” because of its potential to treat diseases like diabetes and cancer and its strong antioxidant potential [22, 23]. BMI and serum selenium deficiency are inversely correlated, and selenium deficiency can cause inflammatory diseases, including obesity. So, due to its property of significantly reducing oxidative stress, is reported to be effective in treating hypertension [24]. Insulin resistance may arise as a result of an increase or decrease in the body's selenium levels. According to research, those with higher selenium levels have a lower risk of getting diabetes than those with lower levels [25, 26].

### 3.2 Chromium (Cr)

As an important trace element, chromium aids in the metabolism of fats and carbohydrates. Promoting the insulin signaling pathway also regulates the amount of insulin and blood glucose. A reduction in chromium levels in the human body causes the blood glucose level to rise. Supplementing with chromium has been shown to be effective in treating metabolic syndrome and insulin sensitivity [27]. It has been reported to up-regulate beta cell sensitivity, insulin sensitivity, and insulin receptors. Research indicates that low amounts of lean body mass and high levels of insulin, cholesterol, triglycerides, and fasting blood glucose are linked to chromium deficiency [28, 29].

### 3.3 Iron

Serum ferritin overload and metabolic syndrome features are associated with each other. Glucose metabolism is affected by iron intake [30]. Increased iron levels in persons with diabetes are at higher risk for cardiovascular disease than healthy individuals. Individuals with the concentration of serum ferritin in Type II diabetes mellitus can affect insulin sensitivity, oxidative damage, viscosity, and vascular resistance. Body mass index and serum ferritin level can act as predictors in glucose tolerance tests. Excess amounts of iron content in the body may lead to severe oxidative stress, which further leads to damage of pancreatic cells and promotes beta cell apoptosis. This will affect the production of insulin, and the risk of insulin resistance will increase. An increase in the level of body iron can increase the risk of obesity and Type 2 diabetes mellitus [31]. It has been reported that iron deficiency anemia can lead to the failure of the heart [32].

### 3.4 Zinc

One of the most prevalent trace elements in the human body, zinc (Zn), functions as a signaling component and is important for growth and development [33]. The relationship between dietary zinc consumption and MetS has been the subject of contradictory findings [34]. Zinc has been shown to reduce oxidative stress by supporting the synthesis of antioxidant enzymes and acting as an enzyme catalyst during the metabolism of carbohydrates, proteins, and lipids [35]. It plays a crucial part in the development of atherosclerosis, type-2 diabetes mellitus, and metabolic syndrome (MS) since it is involved in the production, storage, and release of insulin [36].

### 3.5 Copper (Cu)

The higher serum concentration of copper has been associated with the risk of metabolic syndrome [37]. The risk of non-alcoholic fatty liver disease is substantially increased by elevated serum Cu levels [38, 39]. Copper imbalance can lead to toxic protein aggregation, oxidative stress, mitochondrial malfunction, cellular signaling impairment, inflammation, and amyloid disorders [40]. The concentration of Cu is found to be elevated in type 2 diabetes patients compared to the healthy participants, whereas in diabetic complications, a lower serum level of Cu has been reported. While its exact involvement in the genesis of metabolic syndrome (MetS) is unknown, adult copper levels were positively correlated with abdominal obesity [41].

## 4 DIETARY INTERVENTION

The primary therapeutic approach for the treatment and management of metabolic syndrome is lifestyle change, particularly dietary patterns; however, the most efficient dietary pattern for managing the condition has not yet been determined. Various dietary regimens have been implemented in many parts of the world to address metabolic syndrome and its associated effects. To treat the symptoms of metabolic syndrome, vivid dietary patterns have been outlined below, along with their multifocal aims.

### 4.1 Mediterranean Diet

The effect of MedDiet on various health outcomes has been reported by several systematic reviews and meta-analyses. A variety of health outcomes, including type 2 diabetes, obesity, cancer, decline in cognition, and CVD-related death rates, may be improved by MedDiet, according to research. While MedDiet participants supplemented with nuts only had a significant decrease in abdominal obesity, those supplemented with olive oil saw substantial decreases in both fasting blood sugar and abdominal obesity. A meta-analysis of observational studies found a 19% decrease in MetS risk with higher MedDiet adherence, based on 33,847 participants and 6,342 cases of MetS [42]. However, among people assigned to MedDiet therapies, randomized clinical trials (RCTs) verified the MetS remission. The recommended components of the dietary pattern followed for the Mediterranean diet are represented in Fig. (**2**).

### 4.2 Dietary Approach to Stop Hypertension (DASH) Diet

DASH scores encompass all eight food groups, including grains, meat, vegetables, fruits, low fat-containing dairy products, fats, oils, nuts, legumes, and sweets in whole or part. Moreover, most score versions have a component related to the consumption of salt. MetS is anticipated to be impacted by the DASH diet's notable blood pressure decrease [43-45]. Clinical trials showed that the DASH diet, when combined with lifestyle changes like weight loss, physical exercise, or sodium restriction, efficiently decreases blood pressure across different stages [46]. Irrespective of weight loss, the DASH diet revealed a reduction in fasting insulin concentration & improved insulin sensitivity. There were protective benefits on MetS from dietary patterns that included increased intakes of fruits, dairy, coarse cereals, and soy products [47].

### 4.3 Ketogenic Diet

**Fig. (3):**
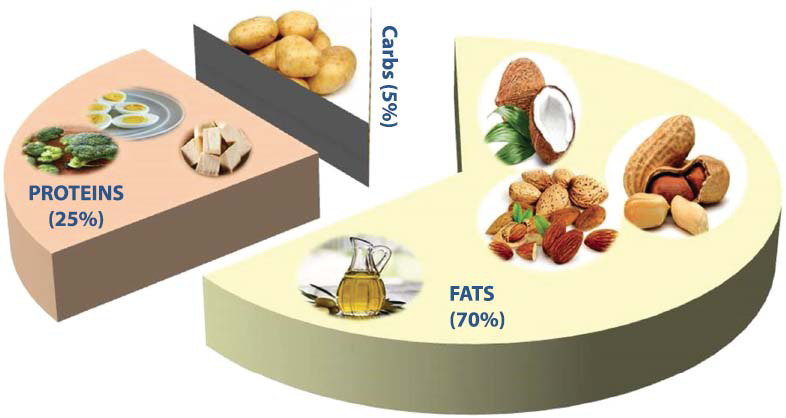
Basic components of the ketogenic diet.

The recent surge in popularity of this diet has prompted scientists to look at how patients' health is affected by this type of eating. Benefits of this diet have been reported, particularly in relation to weight loss [48]. The traditional Ketogenic diet is composed of a combination of fat with proteins and carbohydrates, usually in a ratio of 3:1 or 4:1 (Fig. **3**). The diet aims to achieve ketosis, which may lead to weight loss and improved health outcomes by altering metabolic pathways and lowering blood sugar levels [49, 50]. Even though the meta-analysis revealed a variable response in LDL cholesterol, ketogenic diets have been repeatedly shown to reduce triglyceride levels and raise HDL cholesterol [51]. The ketogenic diet is mainly characterized by a low consumption of dietary carbohydrates, with changing levels of protein & fat [52]. The ketogenic diet is a workable strategy for reducing the likelihood that diabetics will experience difficulties. The amount of insulin needed each day decreased by 67% when a ketogenic diet was adopted [50].

### 4.4 Role of Dietary Fibres

It is important to stress that eating a significant amount of fibre is proven to help with hunger control and weight reduction. A study found that the primary predictor of variance in health outcomes was dietary fibre consumption, indicating that programs aimed at improving body composition and metabolic health should place greater emphasis on ensuring adequate dietary fiber intake [53].

### 4.5 Protein-Rich Diet

Research indicates that increased nuts and legume consumption reduces unhealthy metabolic phenotypes in overweight and obese individuals, possibly influenced by adropin and serum brain-derived neurotrophic factor (BDNF) levels [54]. The gut microbiota benefits from the production of short-chain fatty acids by beneficial gut bacteria, which are supported by the consumption of high-protein foods like peanuts and pistachios. Possible microbiota change in relation to peanuts is associated with reductions in metabolic syndrome risk factors and the inflammatory response induced by a high-fat diet [55].

### 4.6 Pre-Biotic and Probiotic Functional Foods in the Management of Metabolic Syndrome

Microbial variety is undoubtedly crucial to host health, even if it is still unknown exactly what taxonomic makeup creates a “healthy” gut microbiota. Reduced faecal microbial gene richness is linked to a number of physiological indicators of obesity and metabolic syndrome, and obese people have significantly lower bacterial diversity than their lean counterparts [56]. Prebiotic therapy was shown to enhance glucose homeostasis. Additionally, it has been demonstrated that they regulate metabolic abnormalities, particularly those linked to obesity, such as liver steatosis, diabetes, hypertension, and dyslipidemia [57]. By reducing lipopolysaccharide levels, probiotic usage may enhance mucus secretion, boost the population of gut microorganisms, and stop the breakdown of tight junction proteins [58].

## 5 HERBS USED IN THE MANAGEMENT OF METABOLIC SYNDROME

Herbal-based preparation has shown beneficial effects in recovery from MetS-associated symptoms in human subjects. Extracts were found to be useful in reducing triglycerides (TG), high-sensitivity C-reactive protein (hsCRP), low-density lipoprotein cholesterol (LDL-C), and very low-density lipoprotein cholesterol (VLDL-C) in a meta-analysis of *Embilica officinalis* associated randomized controlled trials (RCTs) [59, 60]. A randomized case-control study in Rajasthan found that administering Murraya koenigii extract to 160 hypercholesterolemic urban women resulted in a reduction in central obesity, body weight, BMI, waist circumferences, and visceral fat percentage [61]. A randomized clinical study found that berberine administration reduced metabolic syndrome, waist circumference, SBP, triglycerides, and total insulin secretion while increasing insulin sensitivity [62, 63]. Bergamot juice (*Citrus Bergamia*) significantly decreased blood glucose, triglycerides, total cholesterol, and LDL cholesterol in Metabolic Syndrome patients over six months while increasing HDL cholesterol levels [64, 65]. In a different trial, Chinese patients with dyslipidemia who received a 12-week course of treatment with a multiherb formula containing *Ganoderma lucidum, Crataegus pinnatifida, Stigma maydis, Alisma orientalis, Morus alba,* and *Polygonum multiflorum* showed only modestly positive benefits on plasma LDL-C [66]. In a double-blind, randomized, controlled study including pre-diabetic individuals, it was found that using aloe vera extract might improve blood glucose levels after four weeks, but it could also improve the aberrant lipid profile after eight weeks [67, 68]. The diastolic blood pressure of patients with MetS was dramatically reduced with *Cuminum cyminum* L. essential oil in a randomized, triple-blinded clinical experiment [69]. Additionally, studies have shown that it improves insulin metabolism when compared to a placebo [70-72]. The following table presents research findings on a few commonly used medicinal herbs that have been shown to have some influence on metabolic syndrome in animal studies. The biological models, study design, route of administration (i.p, i.v or orally), and the study outcomes of various herbs in MetS are depicted in Table **1** [73-107].

## CONCLUSION

A healthy dietary strategy appears to have protecting effects on MetS through the combination of small dietary changes rather than a single nutrient restriction. The MedDiet, including low-fat and very-restricted diet supports, is reinforced as a new prototype for MetS prevention and treatment. The low carbohydrate MedDiet style offers easy-to-follow dietary advice for encouraging whole grain and plant-based protein intake in MetS patients. Dietary interventions mainly emphasize weight reduction, frequently through energy-restricted diets, and also improve insulin sensitivity. Daily intake of fiber-rich foods, legumes, low-glycemic foods, nuts, unprocessed cereals, fish, fruit, dairy, and yogurt can prevent and manage the risk of MetS. Additionally, the recommendation is to switch the saturated fatty acids with monounsaturated and polyunsaturated fatty acids and decrease free sugar consumption to less than 10% of total energy intake. Quality of energy-restricted dietary patterns and physical activity are also crucial for managing metabolic disturbances in patients with metabolic syndrome, and this can be easy-to-follow to health professionals. Future developments in diet modifications may provide more insights into genetic features and gut flora.

## Figures and Tables

**Fig. (2):**
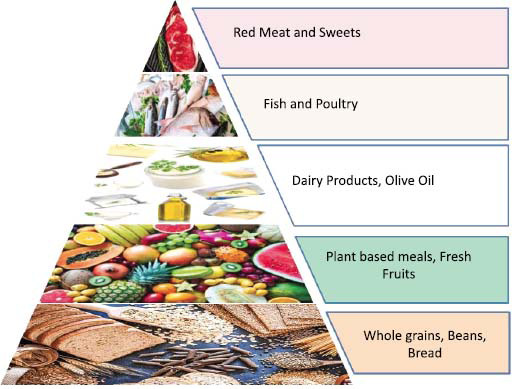
Components of mediterranean diet.

**Table 1:** Herbal extracts with beneficial effects in managing symptoms of metabolic syndrome.

**Name**	**Model/Study Design**	**Dose/ Duration**	**Outcome**	**Reference**
*Emblica officinalis*	Ethyl acetate (EtOAc) extract of amla rich in polyphenols to rats	For two weeks, administered 10 or 20 mg/kg body weight of ethyl acetate extract daily	↓ hypercholesterolemia ↓hypertriacylglycerolemia	[73]
*Murraya koenigii*	Alloxan induced diabetic rats n-18	200 & 500 mg/kg/PO for 1 week	↓ cholesterol and triglyceride levels	[74], [75]
	Streptozotocin-induced diabetes mice	Aqueous extract (75 and 150 mg kg^−^body weight orally once daily) for 15 days.	↓ blood sugar, triglycerides, and total cholesterol; ↑HDL cholesterol	[76]
	HFFD rat model n-36	Aqueous and methanolic extract) for 14 weeks daily at dose level of 200 mg/kg	↓TC and LDL-C. ↑HDL ↓FBGr, TC, LDL, VLDL, and triacylglycerol.	[77], [78]
*Vigna radiate*	HFD fed mice	12 weeks of cooked mung beans (30%, w/w)	↓ LDL-C, triglycerides (TG), and total cholesterol (TC)	[79]
	Diabetic mice	1, 2, 3 g/kg/p. o of mung bean extract	Blocked the dysbiosis of the gut microbiota brought on by the HFD	[80]
	Lean and obese Zucker rats n-40	Food intake weekly for 12 weeks	↓TG and TC; Notable changes to glucose and lipid metabolism in NAFLD	[81]
*Citrus bergamia*	an experimental rat model of MetS n-40	Leaf extract for 10 weeks	↓plasma triglycerides and insulin resistance, ↑ HDL cholesterol	[82]
	Diet-Induced obese rats n-24	Leaves extract for 20 weeks	↓oxidative stress, inflammation, lipid levels in adipose and liver tissues and insulin resistance.	[83]
*Azadirachta indica*	Male rats n-50	For 21 days, 100 and 200 (mg/kg) of methanolic extract were given orally	↓ activities of glutathione peroxidase, superoxide dismutase, and glutathione-S-transferase activity	[84]
	STZ induced diabetes rats	500 mg/kg per day, PO extract for 10 days	↓Cholesterol, TG, BG, LPO; ↑ Anti-oxidant markers protected beta cells and islets Langerhans	[85, 86]
*Crataegus pinnatifida*	Male Kunming mice	300mg/kg concentration/ oral infusion of Fruit for 4 and 10 weeks	↓TG ↓perirenal and epididymal fat, LDL- C;	[87]
	Ovariectomized female Sprague - Dawley rats n-24	For eight weeks, provided 100 mg/kg of the hawthorn extract (OL) or 200 mg/kg of the extract (OH) orally	↓ cholesterol levels, ↓LDL ↓free fatty acids	[88]
*Aloe vera*	Streptozotocin-induced diabetes rats	300-mg/kg Aloe extract for 7 weeks	↓renal oxidative stress, lowering lipid modification, and having a direct renal protective effect.	[89]
	HFD fed Mice n-6	1% w/w Aloe vera powder for a period of 12 weeks	↓ TG, TC, and fat cells	[90]
	Hyperlipidemia induced rats	15 days of 500 mg/kg alcoholic extract of *A. vera*	↓TC, PL, and TG	[91]
*Elettaria cardamomum*	Clinical trial, pre-diabetic women n-80	3g of green cardamom daily for 2 months	↓insulin sensitivity, LDL-C, and TC	[92]
	*In vivo*, hypercholesterolemia induced Wistar rats	3 g/kg, p.o. for 8 weeks	↓in blood total cholesterol, LDL in cholesterol, and serum TG	[93]
	Overweight or obese type 2 diabetic patients n-83	3 g of for 10 weeks	Substantial rise in SIRT1 and a considerable drop in serum HOMA-IR, TG, insulin, and HbA1C	[94]
	Overweight or obese NAFLD patients n-87	Two 500 mg capsules three times per day for 3 months	Notably, decreased levels of fatty liver, high sensitivity CRP (hs-CRP), ALT, IL-6, and TNF-α	[95]
*Zingiber officinale*	Obese Rats fed an HFD	For 30 days,75 mg/kg of gingerol	↓Body mass, blood sugar, and insulin ↓ HMG-CoA reductase activity and mRNA level ↑ mRNA levels and activity of lipoprotein lipase (LPL), lecithin choline acyl transferase (LCAT), and carnitine palmitoyl transferase-1 (CPT-1). ↓inflammatory markers TNF-α and IL-6.	[96]
	Type 2 diabetic model animals, HFD-induced obese Rats	Four weeks, 0.05 percent gingerol The ginger essential oil at dose levels of 12.5, 62.5, and 125 mg/kg and the citral at dose levels of 2.5 and 25 mg per kg were administered for a period of 12 weeks	↓inflammatory markers TNF-α and IL-6. ↑glucose intolerance ↓TG, TC, TBARS, and TNF-α Levels	[97]
*Allium cepa*	High cholesterol diet (HCD)-fed rats	Vegetable extract for six weeks at 8.1g/kg every day	↑Total Lipid in Feces ↓food consumption, TC, HDL, and LDL	[98]
	*In vivo*, rats (obesogenic diet) n-36	for six weeks, quercetin (30 mg/kg/day)	HMG-CoA reductase activity inhibition	[99]
*Brassica oleracea* *Ginkgo biloba*	Rats with alloxan-induced diabetes	300 mg/kg extract for 60 days	↓ Malonaldehyde (MDA), glutathione peroxidase (GPX), and glucose during fasting.	[100]
	Obese ICR Mice Induced by HFD	*B. oleracea* for eight weeks (100 and 200 mg/kg).	↑ insulin resistance, glucose, choline, TG, and leptin levels.	[101]
	Wistar rats under oxidative stress	For three days in a row, take 250 mg/kg twice a day	↑ CAT, ↓GPX and SOD	[102]
	Hepatotoxic and nephrotoxic albino rats caused by gentamicin	Methanolic extract at a dose of 100 mg/kg for 15 days	↑ GPX, MDA, kidney protection, and liver protection	[103]
	Obese mice produced by HFD n-49	broccoli flour (BF) (0.67% or 1.34% w/w) three times a week for 14 weeks	↓weight, build-up of adipose tissue, and ↑ glutathione S-transferase and superoxide dismutase's baseline activities.	[104]
	*In vivo*, rabbits (n-24)	GBE 10 mg/kg p.o. each day for six weeks	Significantly higher GSH and HDL-C and lower TC, TG	[105]
	HFD-induced obese mice	0.1 g/100 g or 0.1% (w/w) for seven weeks	GB improved lipid buildup, decreased blood TG, decreased body weight, and boosted PXR mRNA expression.	[106]
	*In vivo*, rats	Over a 12-week period, daily at dose 100 mg/kg	Markedly lower blood pressure, MDA, nitrite levels, higher mRNA expression of eNOS, protein expressions of iNOS, TNF-α, IL-1β, IL-6 inside kidney, and increased GSH	[107]

## LIST OF ABBREVIATIONS

CRP: C-Reactive Protein
ED: Endothelial Dysfunction
HUVECs: Human Umbilical Vein Endothelial Cells
MetS: Metabolic Syndrome
NO: Nitric Oxide
ROS: Reactive Oxygen Species
Se: Selenium
